# Rho-Kinase/ROCK: A Key Regulator of the Cytoskeleton and Cell Polarity

**DOI:** 10.1002/cm.20472

**Published:** 2010-08-10

**Authors:** Mutsuki Amano, Masanori Nakayama, Kozo Kaibuchi

**Affiliations:** 1 Department of Cell Pharmacology, Graduate School of Medicine, Nagoya University65 Tsurumai, Showa-ku, Nagoya 466-8550, Japan; 2 Department of Tissue Morphogenesis, Max-Planck-Institute for Molecular BiomedicineRoentgenstrasse 20 48149 Muenster, Germany; 3 Japan Science and Technology AgencyCREST, 4-1-8, Honcho, Kawaguchi 332-0012, Japan

**Keywords:** Rho, kinase, cytoskeleton, actomyosin, polarity

## Abstract

Rho-associated kinase (Rho-kinase/ROCK/ROK) is an effector of the small GTPase Rho and belongs to the AGC family of kinases. Rho-kinase has pleiotropic functions including the regulation of cellular contraction, motility, morphology, polarity, cell division, and gene expression. Pharmacological analyses have revealed that Rho-kinase is involved in a wide range of diseases such as vasospasm, pulmonary hypertension, nerve injury, and glaucoma, and is therefore considered to be a potential therapeutic target. This review focuses on the structure, function, and modes of activation and action of Rho-kinase.

## Introduction

Rho-kinase, originally identified as an effector of the small GTPase Rho [Leung et al., 1995; Ishizaki et al., 1996; Matsui et al., 1996], plays a major role in mediating rearrangements of the actomyosin cytoskeleton downstream of Rho. Rho family small GTPases, such as Rho, Rac, and Cdc42, regulate cytoskeletal reorganization in different ways [Kaibuchi et al., 1999; Jaffe and Hall, 2005]. The functions of these GTPases have been primarily investigated with regard to their effects of actin filaments. Rho regulates stress fiber formation and cell contraction, whereas Rac and Cdc42 regulate the formation of lamellipodia and filopodia, respectively, and promote protrusive activities [Hall, 2005]. Rho family GTPases also modulate microtubule dynamics and cell polarity. Furthermore, in addition to Rho-kinase, many other Rho effectors with various functions have been identified, including the myosin phosphatase-targeting subunit 1 (MYPT1) of myosin light chain (MLC) phosphatase, mDia, Protein kinase N (PKN), Citron, Citron-kinase, Rhotekin, and Rhophilin, and many act in concert following Rho activation. For example, Rho-kinase functions together with MYPT1 and mDia to achieve stress fiber and focal adhesion formation downstream of Rho [Amano et al., 2000; Narumiya et al., 2009]. In this review, we will focus on the structure, function, and modes of action of Rho-kinase.

### Structure and Modes of Activation and Inhibition

#### Domain structure

**Fig. 1:**
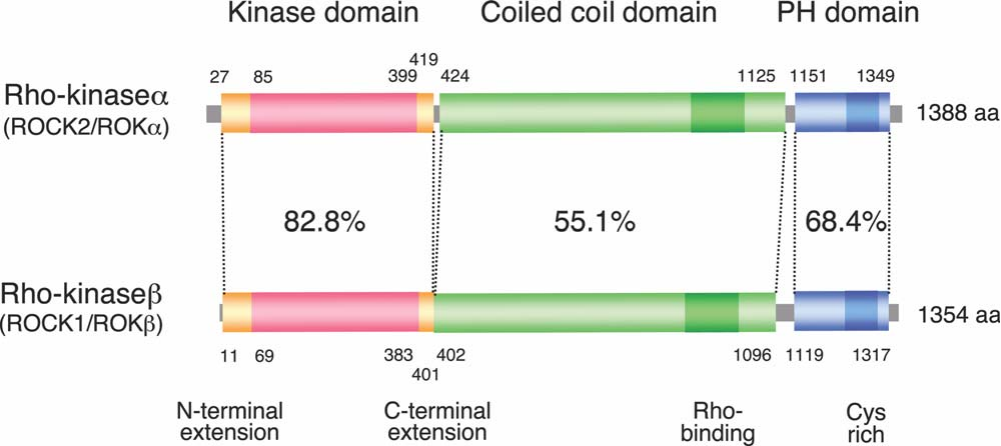
Structure of Rho-kinase/ROCK/ROK Schematic diagrams of the domain structure of Rho-kinases. Amino acid sequence identities for each domain are indicated.

Rho-kinase is a serine/threonine kinase belonging to the AGC family of protein kinases, which are structurally related to myotonic dystrophy kinase (DMPK) and myotonic dystrophy kinase-related CDC42-binding kinase (MRCK). There are two Rho-kinase members, Rho-kinase α/ROCK2/ROKα and Rho-kinase β/ROCK1/ROKβ: we will refer to them collectively as Rho-kinase. Rho-kinase is composed of an N-terminal catalytic domain, a central coiled-coil domain, and a C-terminal PH domain interrupted by a Cys-rich region (Fig. 1). Rho-kinase requires both N- and C-terminal extension segments in addition to the core catalytic domain for its activity, which is conserved among the DMPK subgroup (see below). Rho activates Rho-kinase by binding to the C-terminal portion of the coiled-coil.

#### Crystal structure

**Fig. 2:**
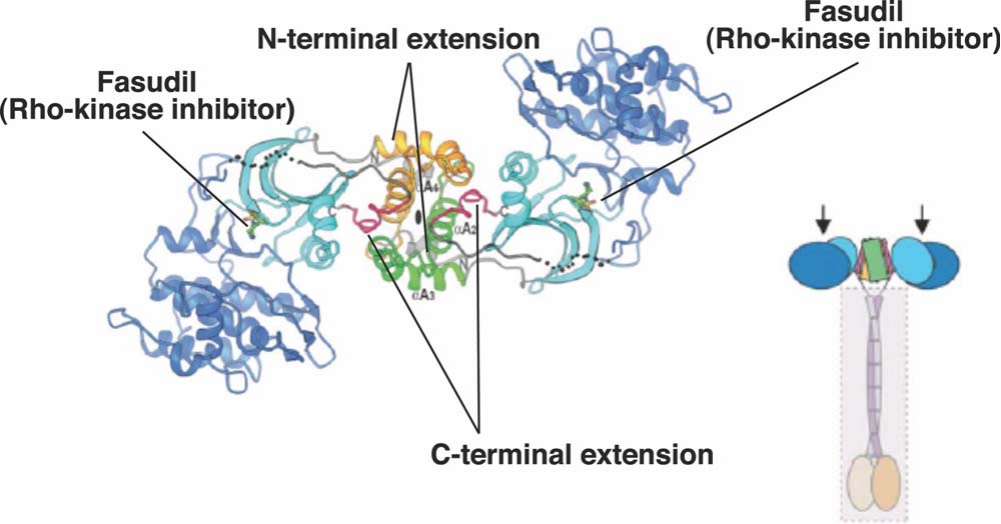
Ribbon diagram of the dimmer structure of catalytic domain of Rho-kinase Rho-kinase forms a head-to head homodimer through its N- and C-terminal extensions. Fasudil binds to the ATP-binding cleft. Predicted whole structure of Rho-kinase is also shown, in which Rho-kinase forms parallel dimmer through both the extensions outside of catalytic domain and central coiled-coil regions. Arrows indicate the active centers of Rho-kinase. Reprinted from Structure, Vol 14(3), 2006, Yamaguchi et al., DOI: 10.1016/j.str.2005.11.024; ©2005, with permission from Elsevier.

Unique features of the Rho-kinase catalytic domain have been revealed by studies of its crystal structures. Rho-kinase has N- and C-terminal extension segments outside the catalytic domain that form an intermolecular head-to-head homodimer, keeping it in an active conformation [Yamaguchi et al., 2006a; Jacobs et al., 2006] (Fig. 2). Interestingly, phosphorylation at the activation loop or C-terminal hydrophobic motif, which is necessary for most of other AGC kinases such as PKC and Akt, is absent from the Rho-kinase catalytic domain in its dimerized active conformation [Yamaguchi et al., 2006a]. The Rho-binding region of Rho-kinase also forms a parallel coiled-coil structure [Shimizu et al., 2003; Dvorsky et al., 2004], supporting the idea that Rho-kinase dimerizes in a parallel manner. Consistent with this, Rho-kinase is found as a multimer in the cell [Chen et al., 2002].

#### Activation and inactivation

The C-terminal segment containing the Rho-binding and PH domains acts as the negative regulatory region of Rho-kinase. That the interaction between active Rho (Rho·GTP) and the Rho-binding domain releases this inhibition is based on several lines of evidence: (1) deletion of the C-terminal portion results in constitutive activation of Rho-kinase [Leung et al., 1996; Amano et al., 1997; Ishizaki et al., 1997], (2) the C-terminal segment interacts with and inhibits the constitutively active catalytic fragment both in vitro and in vivo [Amano et al., 1999], and (3) an antibody against the Rho-binding domain of Rho-kinase enhances its kinase activity in vitro (unpublished observation). In addition to Rho, certain lipids such as arachidonic acid can activate Rho-kinase via its PH domain [Araki et al., 2001]. Proteolytic cleavage at the C-terminus by caspase-3 and granzyme B is also reported to activate ROCK1 and ROCK2, leading to plasma membrane blebbing during apoptosis [Coleman et al., 2001; Sebbagh et al., 2001; Sebbagh et al., 2005]. Other small GTPases besides Rho - Rnd3/RhoE, Gem and Rad - can bind Rho-kinase outside of the Rho-binding region and inhibit its function [Ward et al., 2002; Riento et al., 2003; Komander et al., 2008]. It has also been reported that PDK1 directly binds to ROCK1 in competition with RhoE, leading to the release of ROCK1 from RhoE and activation [Pinner and Sahai, 2008].

#### ROCK1 and ROCK2

The structures of ROCK1 and ROCK2 are conserved with 64% overall amino acid identity (Fig. 1). The kinase domain containing both extension segments is more highly conserved between these two proteins (83% identical), suggesting that they may have similar substrate specificity. The consensus phosphorylation sequence for Rho-kinase is R/KXS/T or R/KXXS/T (X is any amino acid). Both Rho-kinase proteins are ubiquitously expressed in most tissues; however, higher levels of ROCK2 are found in brain and muscles whereas higher levels of ROCK1 are found in non-neuronal tissues including liver, lung and testis [Leung et al., 1996; Nakagawa et al., 1996]. So far, several functional differences between ROCK1 and ROCK2 have been reported. ROCK1 is specifically cleaved by caspase-3, whereas ROCK2 is cleaved by granzyme B [Coleman et al., 2001; Sebbagh et al., 2001; Sebbagh et al., 2005]. RhoE preferentially binds ROCK1, but not ROCK2, whereas MYPT1 binds only ROCK2 [Komander et al., 2008; Wang et al, 2009]. Gene silencing experiments also suggest different cellular functions for these two proteins, ROCK1 appears to be essential for the formation of stress fibers, whereas ROCK2 appears to be necessary for phagocytosis and cell contraction, both of which are dependent on MLC phosphorylation [Yoneda et al., 2005; Wang et al., 2009]. Most if not all mice carrying a homozygous deletion of the either the ROCK2 or ROCK1 allele show embryonic or postnatal lethality, respectively, whereas heterozygous mice are fertile and appear normal [Thumkeo et al., 2003; Shimizu et al., 2005; Thumkeo et al., 2005]. However, the question of whether these two Rho-kinase proteins are qualitatively different or expressed at different abundances in tissues remains unanswered.

#### Inhibitors

To elucidate the physiological roles of Rho-kinase, small molecule inhibitors have been developed and investigated in various cell types and animal models [Mueller et al., 2005; Shimokawa and Rashid, 2007]. Fasudil (HA-1077) and Y-27632 have been broadly used as Rho-kinase selective inhibitors and function in an ATP-competitive manner. Fasudil, composed of the isoquinoline ring and the pendant ring of the seven-membered homopiperazine, is used clinically for cerebral vasospasm after subarachnoid hemorrhage in Japan. H-1152 has been developed by optimization of a series of isoquinoline compounds. Y-27632 was identified by its ability to inhibit phenylephrine-induced contraction of a rabbit aortic strip and contains a 4-aminopyridine ring [Uehata et al., 1997]. Structural analyses of the Rho-kinase (ROCK2)-Fasudil complex [Yamaguchi et al., 2006a], Rho-kinase (ROCK2)-Y-27632 complex [Yamaguchi et al., 2006b], and ROCK1-Y-27632 complex [Jacobs et al., 2006] revealed the inhibitors cause an induced-fit conformational change to increase contacts with Rho-kinase phosphate loop, which may account for their specificity. Several pharmaceutical companies are investing in the development of Rho-kinase inhibitors for the treatment of certain diseases, such as glaucoma (see [Hahmann and Schroeter, 2010] for a review). Notably, the ROCK2-selective inhibitor SLx-2119 [Boerma et al., 2008] is now in clinical trials.

### Functions

**Fig. 3:**
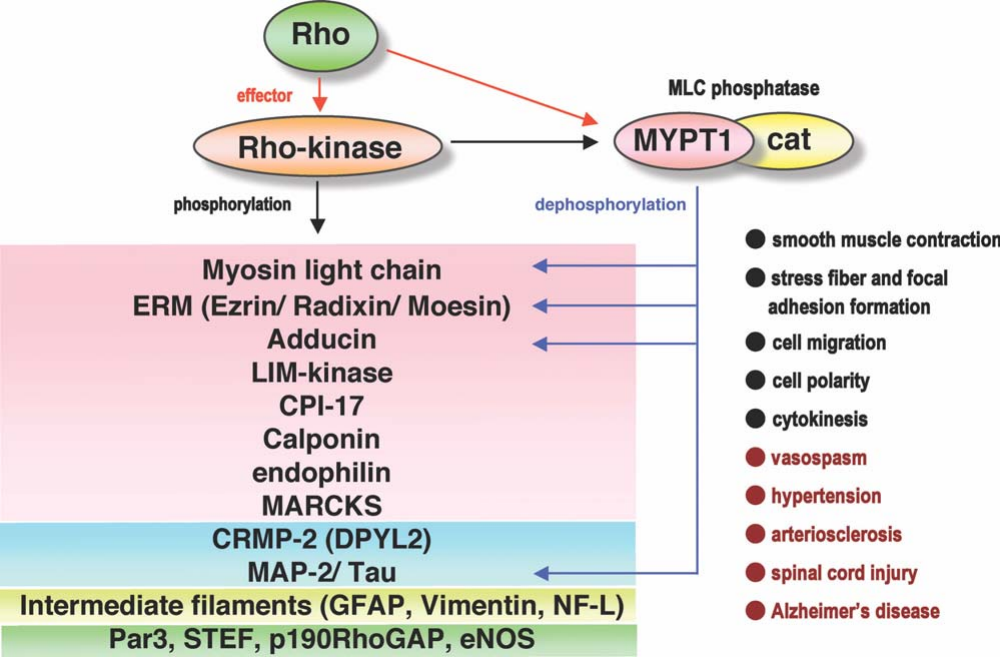
Substrates of Rho-kinase Rho-kinase inhibits the MLC phosphatase activity through both phosphorylation of MYPT1 of MLC phosphatase and phosphorylation of CPI17, an inhibitory protein of myosin phosphatase. Rho-kinase and MLC phosphatase share their substrates, such as MLC, ERM proteins, adducin and MAPs, and thought to regulate the level of phosphorylation. The substrates reported to be phosphorylated by Rho-kinase are illustrated; actin-bindig/regulating proeins (red), MT-binding/regulating proteins (blue), intermediate filaments (yellow), and proteins in other signaling pathways (green). Physiological (black) and pathological (red) processes in which Rho-kinase is involved are listed at the bottom right.

Rho-kinase members play roles in various cellular functions through phosphorylation of their specific substrates (Fig. 3 and Table I)

**Table I:** List of Known Substrates and Effects of Phosphorylation

Substrate	Effect of phosphorylation	Cellular function	Reference
Microfilament			
MYPT1	Inhibition of MLC phosphatase	Increase in cell contraction	[Kimura et al., 1996]
MLC	Activation of myosin ATPase	Increase in cell contraction	[Amano et al., 1996]
ERM	Maintenance of crosslinking	Microvilli formation?	[Fukata et al., 1998] [Oshiro et al., 1998] [Matsui et al., 1998]
Adducin	Enhancement of F-actin binding	Cell migration	[Fukata et al., 1999]
LIMK1/2	Activation of cofilin phosphorylation	Stress fiber formation	[Maekawa et al., 1999] [Ohashi et al., 2000]
Calponin	Inhibition of F-actin binding	n.d.	[Kaneko et al., 2000]
CPI-17	Inhibition of MLC phosphatase	Increase in cell contraction	[Koyama et al., 2000]
NHE1	n.d.	Stress fiber formation	[Tominaga and Barber, 1998] [Denker et al., 2000]
MARCKS	Inhibition of F-actin binding	n.d.	[Nagumo et al., 2001]
EF1α	Inhibition of F-actin binding	n.d.	[Izawa et al., 2000]
Troponin I/T	n.d.	Inhibition of Ca2+ response in cardiac muscle	[Vahebi et al., 2005]
Profilin	Inhibition of G-actin binding	Decrease in polyQ aggregation	[Shao et al., 2008] [Bauer et al., 2009]
Microtubule			
MAP2/Tau	Inhibition of tubulin polymerization	Inhibition of neurite elongation?	[Amano et al., 2003]
CRMP2	Inhibition of tubulin polymerization	Growth cone collapse	[Arimura et al., 2000; Arimura et al., 2005]
Doublecortin	Inhibition of microtubule bundling	Neuronal migration?	[Amano et al., 2010]
Intermediate filament			
GFAP	Depolymerization	Progression of cytokinesis	[Yasui et al., 1998]
Vimentin	Depolymerization	Progression of cytokinesis	[Goto et al., 1998]
Neurofilament (NF-L)	Depolymerization	Neurite retraction	[Hashimoto et al., 1998]
Signaling crosstalk			
Par3	Dissociation of Par3/Tiam1 from Par6/aPKC	Control of cell migration	[Nakayama et al., 2008]
STEF	Inhibition of GEF activity?	Inhibition of neurite outgrowth?	[Takefuji et al., 2007]
p190RhoGAP	Inhibition of Rnd binding	Positive feedback signal of Rho?	[Mori et al., 2009]
eNOS	Inactivation of eNOS	Cell contraction?	[Sugimoto et al., 2007]
PTEN	Activation of phosphatase activity?	Cell polarity?	[Li et al., 2005]
FilGAP	Activation of RacGAP activity	Lamella formation, cell polarity	[Ohta et al., 2006]
IRS1		Positive/negative regulation of insulin signal?	[Furukawa et al., 2005]
Endophilin	Inhibition of CIN85 binding	Inhibition of EGFR endocytosis	[Kaneko et al., 2005]
P300	Increase in acetyltransferase activity	Enhancement of transcription	[Tanaka et al., 2006]
RhoE	Stabilization	Decrease of stress fibers	[Komander et al., 2008; Riento et al., 2003]

n.d.: not defined.

#### Cell contraction and actin organization

##### Smooth muscle contraction

Vascular smooth muscle tone is regulated by MLC phosphorylation of myosin II, which is mediated by Ca^2+^-dependent MLC kinase and MLC phosphatase. The Rho/Rho-kinase signaling pathway is known to modulate the “Ca^2+^-sensitivity” of smooth muscle mainly through the suppression of MLC phosphatase activity. MLC phosphatase is composed of the catalytic subunit (PP1cδ) and two regulatory subunits, myosin phosphatase-targeting subunit 1 (MYPT1) and M20 [Ito et al., 2004]. Rho-kinase phosphorylates MYPT1 at two inhibitory sites, Thr853 and Thr696, resulting in a decrease in MLC phosphatase activity and an increase in phosphorylated MLC [Kimura et al., 1996]. Rho-kinase can also phosphorylate MLC at Ser19 to increases myosin ATPase activity at least in vitro [Amano et al., 1996], although a direct contribution of Rho-kinase to phospho-MLC levels in vivo is not yet proved. CPI-17 has an inhibitory effect on MLC phosphatase when it is phosphorylated by PKC at Thr38 and is also reported to be phosphorylated by Rho-kinase at the same site [Kitazawa et al., 2000] [Koyama et al., 2000]. Several studies using permeabilized smooth muscle and selective inhibitors have shown that Rho-kinase activity is involved in the modulation of smooth muscle tone, especially during aberrant vascular contractions, such as coronary vasospasm, cerebral vasospasm after subarachnoid hemorrhage, and pulmonary hypertension [Shimokawa and Rashid, 2007]. Recently, we found that Rho-kinase phosphorylates p190RhoGAP, one of the major negative regulators of Rho, and presumably inhibits its GAP activity in smooth muscle cells [Mori et al., 2009], which may form a positive feedback loop that accounts for the hyperactivation of Rho at spastic sites.

##### Stress fiber formation

Stress fibers and focal adhesions confer contractility on a cell and are mediated by Rho/Rho-kinase signaling pathway [Amano et al., 1997]. MLC phosphorylation which is necessary for the formation and maintenance of stress fibers and focal adhesions, is regulated by Rho-kinase and MLC phosphatase downstream of Rho, in cooperation with the Rho effector mDia [Watanabe et al., 1999]. LIM kinases 1 and 2 are phosphorylated by Rho-kinase at Thr508 and Thr505, respectively, resulting in increased cofilin phosphorylation at Ser3 [Maekawa et al., 1999]. Cofilin is an actin-depolymerizing factor and regulates actin dynamics, and its activity is inhibited by phosphorylation. Rho-kinase seems to induce and maintain stress fibers by increasing contractility via MLC phosphorylation and by stabilizing actin filaments through LIMK activation which results in cofilin phosphorylation. Isolated contractile stress fibers from fibroblasts contain Rho, Rho-kinase and MYPT1, and their contraction is sensitive to Rho-kinase inhibitors [Katoh et al., 2001], suggesting that Rho, Rho-kinase and MLC phosphatase associate with microfilaments to form a modulatory apparatus for acto-myosin contraction.

#### Cell migration

##### Mode of cell migration

Multiple events are coordinately regulated during cell migration: polarization in the direction of movement, reorganization of the cytoskeleton, turnover of membranes, and remodeling of cell adhesions. Cell-to-cell and cell-to-substratum interactions are important for cell migration. Proper cell movement is essential for development, wound healing and inflammation. Aberrant cell migration may lead to increased tumor cell invasion and arteriosclerosis. Rho family GTPases play critical roles in regulation of cell migration through their specific effectors [Etienne-Manneville and Hall, 2002; Fukata et al., 2003].

The roles of Rho/Rho-kinase in cell migration depend on the cell type and mode of migration [Nakayama et al., 2005]. Inhibition of Rho or Rho-kinase results in the inhibition of migration through Boyden chambers, but not attachment, of monocytes [Worthylake et al., 2001], neutrophils, HL-60 [Hauert et al., 2002], eosinophils [Alblas et al., 2001], and smooth muscle cells [Ai et al., 2001; Nishiguchi et al., 2003]. Monocytes treated with Y-27632 or C3 exoenzyme (an inhibitor of Rho) have elongated tails, and cause a defect in tail detachment, and a mislocalizated integrins, the latter of which is not observed in cells treated with myosin inhibitors [Worthylake et al., 2001]. This suggests that not only myosin-mediated contraction but also integrin reorganization is necessary for the Rho/Rho-kinase-mediated tail retraction. Rho or Rho-kinase inhibitors also affect the chemokinesis of neutrophils and HL-60 cells. fNLPNTL-induced neutrophil polarization is inhibited to some extent by treatment with Y-27632, whereas the number of non-polar cells with ruffles is increased [Hauert et al., 2002]. Similar results have been shown for HL-60 cells stimulated with fMLP - the number of cells with flattened morphologies and multiple pseudopods is increased by the inhibition of Rho, Rho-kinase or myosin II [Xu et al., 2003]. In Y-27632-treated HL-60 cells, the level of activated Rac is increased after stimulation with fMLP, suggesting that the multiple pseudopods observed after the inhibition of Rho/Rho-kinase are the consequence of aberrant activation of Rac. The migration of fibroblasts during wound healing or random migration is not inhibited, but rather enhanced, by inhibition of Rho or Rho-kinase [Nobes and Hall, 1999; Magdalena et al., 2003; Totsukawa et al., 2004]. In contrast to neutrorphils and HL-60 cells, Rho-kinase inhibition by Y-27632 leads to faster and straighter migration with a single leading edge and without Rac activation, whereas MLCK inhibition results in the formation of multiple protrusions in gerbil fibroma cells [Totsukawa et al., 2004].

Furthermore, it has been shown that tumor cells use at least two types of migration in a three-dimensional matrix: Rho/Rho-kinase-dependent and Rac-dependent migration [Sahai and Marshall, 2003]. Several tumor cell lines, such as A375 melanoma cell lines, can migrate through a 3D matrix in a Rac-dependent mesenchymal fashion or in a Rho/Rho-kinase-dependent amoeboid fashion. Inhibition of Rho-kinase converts their morphology from amoeboid to mesenchymal in a DOCK3 (RacGEF)/Rac/WAVE2-dependent manner, whereas silencing of DOCK3 or Rac1 converts cells from the mesenchymal morphology to amoeboid one in a Rho-kinase/ARHGAP22 (RacGAP)-dependent manner, suggesting that these two types of migration are interconvertible and antagonistic [Sanz-Moreno et al., 2008].

##### Front-rear polarity

The establishment of front-rear polarity is another important process for efficient cell migration. Various molecules and/or activation states of proteins are distributed asymmetrically in the polarized cell. Rho-kinase inhibitors impair cell polarization, leading to random protrusions and multiple leading edges in certain cell types. This suggests that Rho-kinase is necessary for breaking symmetry and/or maintaining the polarized imbalance, by suppressing extension of membrane except at the front leading edge. In neutrophils, PTEN is localized at the rear of cell and suppresses the production of PI(3,4,5)P_3_; the distribution of PTEN is under the control of Rho/Rho-kinase, most likely by the phosphorylation of PTEN by Rho-kinase [Li et al., 2005]. We recently showed that Rho-kinase also phosphorylates PAR-3, a component of the PAR polarity complex, which leads to dissociation of PAR-3 from the PAR-6/aPKC complex [Nakayama et al., 2008]. Because PAR-3 binds to the Rac GEF Tiam1/Tiam2 (STEF) and aPKC stimulates Rac GEF activity, Rho-kinase indirectly abrogates Rac activation by phosphorylating PAR-3. PAR-3 phosphorylation by Rho-kinase is observed not only at the rear of the cell but also at the leading edge, which may reflect the role of Rho-kinase in modulating the extent of protrusions by counteracting Rac activity.

#### Neurite elongation and neuronal architecture

##### Growth cone collapse

Rho and Rho-kinase are highly expressed in the nervous system, implying important roles for these proteins in neuron and/or glial cells. Several neuron-specific substrates for Rho-kinase have been identified. Collapsin response mediator protein 2 (CRMP-2) is abundant in brain tissue, and is implicated in growth cone collapse and neuronal polarization through its interaction with tubulin heterodimers, numb, kinesin-1, and Sra-1. CRMP-2 is phosphorylated by Rho-kinase at Thr555, which impairs its ability to bind to tubulin and numb [Arimura et al., 2005]. Phosphorylation of CRMP-2 is increased by stimulation with LPA and ephrin-A5 in DRG and hippocampal neurons, respectively, and is implicated in growth cone collapse induced by these repulsive cues [Arimura et al., 2000; Arimura et al., 2005]. MAP2, Tau, and neurofilament are also neuron-specific substrates for Rho-kinase, and their microtubule polymerizing activity and neurofilament assembly are inhibited by phosphorylation by Rho-kinase [Hashimoto et al., 1998; Amano et al., 2003]. Phosphorylation of MAP2/Tau and neurofilament may prevent neurite elongation and shorten neurites by destabilizing microtubules and intermediate filaments. The control of cellular contraction through MYPT1/MLC phosphorylation by Rho-kinase is also thought to participate in neurite retraction and inhibition of neurite outgrowth [Amano et al., 1998; Hirose et al., 1998]. LIMK1/cofilin has also been implicated in regulation of growth cone morphology in chick DRG neurons [Endo et al., 2003], potentially downstream of Rho-kinase.

##### Neuronal architecture and neurite elongation

Pharmacological studies have revealed many roles for Rho-kinase in the nervous system. Treatment of cultured cerebellar granule neurons with Y-27632 triggered immediate neurite outgrowth at an early stage, though it was less effective at later stages, suggesting that Rho-kinase controls the initiation of axonogenesis [Bito et al., 2000]. At the neuronal maturation stage, dendritic branching and spine formation are markedly inhibited when constitutively activated RhoA is expressed in hippocampal slice cultures; this effect can be abrogated by the administration of Y-27632 [Nakayama et al., 2000], implying that Rho-kinase plays a role in dendrite formation.

Rho-kinase inhibitors have also been shown to be effective for treatment of some neurological disorders, including spinal cord injury and Alzheimer's disease [Mueller et al., 2005]. Several myelin-derived proteins are known to prevent axon regeneration after axon damage in the central nervous system. Myelin-associated inhibitors, such as Myelin-associated glycoprotein (MAG), Nogo, and oligodendrocyte myelin glycoprotein (OMgp) activate Rho through the p75 receptor in complex with co-receptors, and Rho-kinase inhibitors abolish myelin-associated inhibitor-induced repellant effects in both cultured neuron and animal models [Mueller et al., 2005]. One of the potential downstream effectors of Rho-kinase in these signaling pathways is CRMP-2. CRMP-2 is phosphorylated at Thr555 following treatment with MAG or Nogo, and overexpression of CRMP-2 causes neurite extension in the presence of MAG [Mimura et al., 2006].

Rho-kinase inhibitors also show a potential protective effects in Alzheimer's disease [Tang and Liou, 2007]. Alzheimer's disease is characterized by extracellular amyloid aggregates of toxic 40- or 42-amino-acid long amyloid-β (Aβ40 or Aβ42) peptides, which are generated from amyloid precursor protein (APP) that is abnormally cleaved by α- and γ-secretases. Treatment with Y-27632 decreased the production of Aβ42 in SH-SY5Y cells expressing mutant APP (APPswe) and in model mice [Zhou et al., 2003]. Shedding of APP, which determines the alternative forms, soluble APP (sAPP) and toxic Aβ42, was also found to be modulated by Rho-kinase in APPswe-expressing N2a cells [Pedrini et al., 2005].

#### Cytokinesis

Drastic reorganization of the cytoskeleton occurs during cell division. During cytokinesis, cells are divided into two daughter cells via an actomyosin-based contractile apparatus known as the contractile ring to which Rho and Rho-kinase are recruited. In addition to Rho, Plk1 has also been found to associate with and activate Rho-kinase synergistically with Rho during cytokinesis [Lowery et al., 2007]. Rho and Rho-kinase are involved in both the progression of the cleavage furrow formation and the disassembly of intermediate filaments beneath cleavage furrow [Matsumura, 2005] [Izawa and Inagaki, 2006]. Rho-kinase regulates MLC phosphorylation through MLC phosphatase, and possibly by direct phosphorylation, at the contractile ring. At the same time, Rho-kinase phosphorylates the head domain of intermediate filaments, such as vimentin, leading to the disassembly of filaments that ensures furrow completion. Inhibition of Rho-kinase by Y-27632 does not completely arrest cytokinesis in cultured cells, though it does induce some abnormalites and delays in cleavage [Kosako et al., 2000]. Various other kinases, such as Aurora-B and Citron-kinase, seem to play redundant roles in cytokinesis.

#### Other functions

Ezrin/radixin/moesin (ERM) proteins were originally identified as Rho-kinase substrates that are phosphorylated at Thr567, Thr564, and Thr558, respectively; phosphorylation of ERM proteins by Rho-kinase maintains their activity by crosslinking transmembrane proteins to F-actin [Fukata et al., 1998; Matsui et al., 1998]. PKC and MRCK were also demonstrated to phosphorylate ERM at these sites, and the contribution of Rho-kinase to ERM phosphorylation is thought to depend on the cell type and situation, such as in smooth muscle cells upon static pressure [Onoue et al., 2008], in hippocampal neurons upon glutamate stimulation [Jeon et al., 2002], in T cells from systemic lupus erythematosus patients [Li et al., 2007], and in Jurkat cells upon Fas ligand stimulation [Hebert et al., 2008].

Rho-kinase is also localized in the nucleus and interacts with p300 acetyltransferase, which forms a large nuclear protein complex [Tanaka et al., 2006]. Rho-kinase phosphorylates p300 and activates its acetyltransferase activity, in vitro and in vivo, resulting in the enhancement of p300-dependent transcription in U205 cells [Tanaka et al., 2006].

Many genetic and pharmacological studies have implicated Rho-kinase in various cellular functions in which the key substrates remain obscure. Rho/Rho-kinase has been demonstrated to be involved in planar polarity establishment downstream of the Wnt/Frizzled/Dishevelled pathway in Drosophila eye and wing development [Winter et al., 2001] and in zebrafish gastrulation convergence and extension [Marlow et al., 2002]. Nucleophosmin (NPM)/B23 is a centrosomal protein that is reported to associate with Rho-kinase at the centrosome [Ma et al., 2006]. NPM activates Rho-kinase in a phosphorylation state-dependent manner by CDK2/cyclin E, which is involved in preventing centrosome reduplication [Ma et al., 2006]. Several lines of evidence suggest that the Rho-kinase signaling pathway also modulates cell survival and apoptosis, as Rho-kinase is known to be involved in morphological changes that occur during apoptosis [Coleman et al., 2001; Sebbagh et al., 2001; Sebbagh et al., 2005]. Recently, Rho-kinase inhibitors such as Y-27632 have been shown to have protective effects on human embryonic stem cells. Administration of Y-27632 prevents apoptosis and enhances survival of human embryonic stem cells at low culture density [Watanabe et al., 2007], possibly through the regulation of MLC phosphorylation and cell-cell interactions [Harb et al., 2008]. However, inhibition of Rho-kinase activity can promote apoptosis in certain situations [Shibata et al., 2003; Svoboda et al., 2004; Moore et al., 2004] and enhances cytotoxic polyglutamate (polyQ) aggregates of huntingtin, androgen receptor, ataxin-2, and atropin-1 [Shao et al., 2008; Bauer et al., 2009].

### Future Directions

Intensive studies of Rho and Rho-kinase have revealed their importance in diverse cellular processes and pathologies, paticularly in cultured cell lines, the cardiovascular system, and the nervous system. One of the critical roles of Rho-kinase is modulation of cellular contractility, and aberrant activation of Rho-kinase appears to be involved in some cardiovascular and neurological diseases. However, the functions of Rho-kinase in other tissues still remain largely unsolved, despite its nearly ubiquitous expression in the body. The generation and study of conditional knock-out and knock-in mice of two family members of Rho-kinase would provide important insight into their physioogical roles. Furthermore, a comprehensive identification of Rho-kinase substrates is necessary for better understanding of Rho-kinase signaling networks. We have recently identified more than 100 potential substrates of Rho-kinase by the interactome approach, using the catalytic domain of Rho-kinase, and have isolated novel substrates such as Doublecortin, AP180 and APP [Amano et al., 2010]. Rho-kinase contributes to complicated intracellular signaling networks in a wide range of situations, and further analysis will shed light on its biological mechanisms and potential therapeutics for the disease treatment.
